# Docking, Interaction Fingerprint, and Three-Dimensional Quantitative Structure–Activity Relationship (3D-QSAR) of Sigma1 Receptor Ligands, Analogs of the Neuroprotective Agent RC-33

**DOI:** 10.3389/fchem.2019.00496

**Published:** 2019-07-11

**Authors:** José Luis Velázquez-Libera, Giacomo Rossino, Carlos Navarro-Retamal, Simona Collina, Julio Caballero

**Affiliations:** ^1^Centro de Bioinformática y Simulación Molecular, Facultad de Ingeniería, Universidad de Talca, Talca, Chile; ^2^Pharmaceutical and Medicinal Chemistry Section, Drug Sciences Department, Università di Pavia, Pavia, Italy

**Keywords:** sigma1 receptor ligands, RC-33, arylalkylamine derivates, docking, quantitative structure–activity relationships, interaction fingerprints

## Abstract

The human Sigma1 receptor (S1R), which has been identified as a target with an important role in neuropsychological disorders, was first crystallized 3 years ago. Since S1R structure has no relation with another previous crystallized structures, the presence of the new crystal is an important hallmark for the design of agonists and antagonists against this important target. Some years ago, our group identified RC-33, a potent and selective S1R agonist, endowed with neuroprotective properties. In this work, drawing on new structural information, we studied the interactions of RC-33 and its analogs with the S1R binding site by using computational methods such as docking, interaction fingerprints, and receptor-guided alignment three dimensional quantitative structure–activity relationship (3D-QSAR). We found that RC-33 and its analogs adopted similar orientations within S1R binding site, with high similitude with orientations of the crystallized ligands; such information was used for identifying the residues involved in chemical interactions with ligands. Furthermore, the structure-activity relationship of the studied ligands was adequately described considering classical QSAR tests. All relevant aspects of the interactions between the studied compounds and S1R were covered here, through descriptions of orientations, binding interactions, and features that influence differential affinities. In this sense, the present results could be useful in the future design of novel S1R modulators.

## Introduction

The Sigma receptors (SR) have attracted the interest of the scientific community thoroughly in the last decades owing to their potential role in cell survival and function modulation (Walker et al., 1990; Chu and Ruoho, 2016). They were originally misclassified as a subtype of opioid receptors (Martin et al., 1976), but they were later classified as unique class of intracellular proteins, distinct from other receptors such as GPCRs (G protein-coupled receptors). Sigma receptors (SRs), comprise two subtypes σ_1_ and σ_2_ receptors (S1R and S2R, respectively) associated with aging- and mitochondria-associated disorders (Tesei et al., 2018). Both subtypes are highly expressed in the central nervous system, but they are derived from completely different genes. S1R was cloned in 1996 (Hanner et al., 1996) and was crystallized for the first time 3 years ago, in 2016 (Schmidt et al., 2016), whereas S2R was cloned only very recently, in 2017, by Alon et al. (2017).

S1R is an intracellular modulator between the endoplasmic reticulum and the mitochondria, the cell nuclei, the membrane, and it also modulates intracellular signaling. It plays a key role in neuropsychological disorders such as depression, enhances the glutamatergic neurotransmission (DeCoster et al., 1995; Meyer et al., 2002), and modulates second messenger systems, such as the phospholipase C/protein kinase C/inositol 1,4,5-trisphosphate system (Morin-Surun et al., 1999). Multiple biological roles of S1R have been identified, which made this protein a relevant target for the future treatment of epilepsy, schizophrenia, sclerosis, Alzheimer, and Parkinson's diseases, cancer, etc. (Mishina et al., 2005; Hashimoto, 2009; Furuse and Hashimoto, 2010; Mavlyutov et al., 2015; Vavers et al., 2017; Tesei et al., 2018). Moreover, S1R agonists enhanced neuroplasticity, and may be effective in amyotrophic lateral sclerosis (Peviani et al., 2014) and multiple sclerosis (Collina et al., 2017b).

Not less important, preclinical studies carried out on different models of memory impairment have revealed that S1R ligands could be promising drugs to treat cognitive dysfunctions (Hayashi and Su, 2004; Monnet and Maurice, 2006; Yagasaki et al., 2006; Collina et al., 2017a). Therefore, the identification of potent and selective S1R modulators is of great interest to develop novel therapeutic strategies focused mainly in the treatment of central nervous system disorders. The list of S1R ligands in the last years includes thioxanthene-derived compounds (Glennon et al., 2004), fenpropimorph-derived analogs (Hajipour et al., 2010), 2(3*H*)-benzothiazolones (Yous et al., 2005), cyclopropylmethylamines (Prezzavento et al., 2007), benzo[d]oxazol-2(3H)-one derivatives (Zampieri et al., 2009), etc. All these compounds were developed when the three-dimensional (3D) structure of S1R was unknown. Despite this, the pharmacophoric features of S1R were identified and these compounds comply with the general accepted pharmacophoric pattern. It was demonstrated that at least one N positively charged atom is important for binding at sigma receptors and this atom must be flanked by two hydrophobic regions of different sizes (Ablordeppey et al., 2000; Glennon, 2005; Caballero et al., 2012).

In the last years, we designed and synthesized compounds that comply with the proposed pharmacophore model and evaluated them as S1R ligands (Collina et al., 2007; Urbano et al., 2007; Rossi et al., 2010, 2011), leading to the finding of compound RC-33 as a potent and selective S1R agonist (Rossi et al., 2013a; Marra et al., 2016). The structure-activity relationship (SAR) of the majority of these compounds was previously described by us by using 2D-QSAR methodologies (Quesada-Romero et al., 2015). With the recent report of the S1R 3D structure (Schmidt et al., 2016), structure-based molecular modeling methods could be used to investigate S1R ligands with a new glance. With this in mind, we propose in this work the analysis of the SAR of RC-33 and its analogs (in total there were 80 compounds) by combining docking and a 3D-QSAR methodology. This is the first study focused on describing the SAR of S1R ligands by using structure-based molecular modeling methods, after the report of the crystallographic structure of this important biological target.

## Materials and Methods

### Dataset Preparation

The studied compounds were extracted from references (Collina et al., 2007; Urbano et al., 2007; Rossi et al., 2010, 2011, 2015, 2017; Rui et al., 2016). This dataset yielded a total of 80 compounds with reported activities as Ki ranging from 0.00069 to 1 μM. Ki values were converted into logarithmic pKi values prior 3D-QSAR models' elaboration. The compound chemical structures and their pKi values are depicted in Table 1. The molecular structures were sketched using Maestro's molecular editor (Maestro 10.2.011, Schrödinger LLC). Thereafter, the 3D structures were obtained with the help of the LigPrep module (LigPrep, Maestro 10.2.011, Schrödinger LLC); ionization states were generated at pH 7.0 ± 2.0 using Epik (Shelley et al., 2007). For compounds containing two possible enantiomers which are reported in racemic form, the *R* enantiomer was chosen for QSAR experiments because it was determined that both RC-33 enantiomers showed similar affinities for the S1R and they are almost equally effective as S1R agonists (Rossi et al., 2013b). However, both enantiomers were chosen for docking experiments to explore the interactions in the S1R binding site.

**Table 1:** Structures of RC-33 analogs as S1R ligands.

**ID**	**Structure**	**Experimental pKi^b^**	**Predicted pKi^b^**	**References**
1 (RC-33)	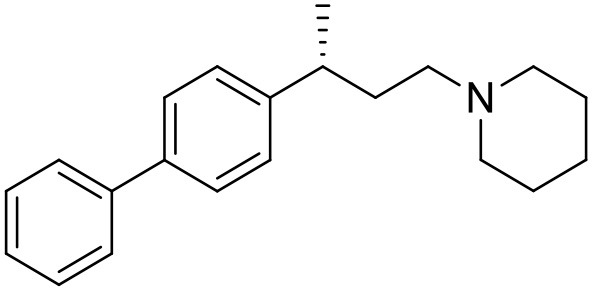	9.16	9.37	Rossi et al., 2011
2	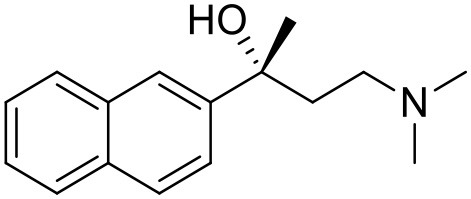	7.60	7.54	Collina et al., 2007
3	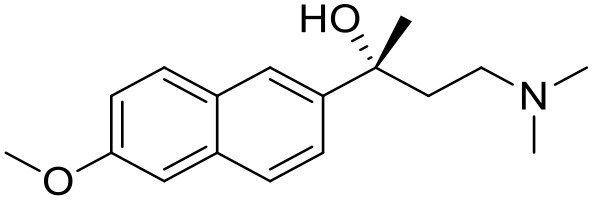	7.41	7.49	Rossi et al., 2010
4	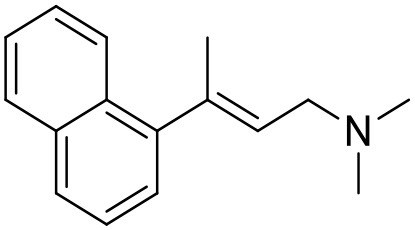	6.99	6.96	Collina et al., 2007
5	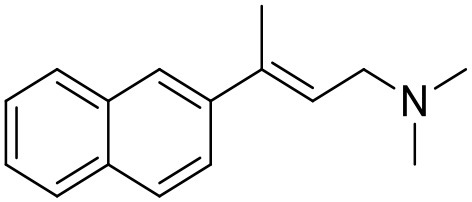	7.70	7.62	Collina et al., 2007
6	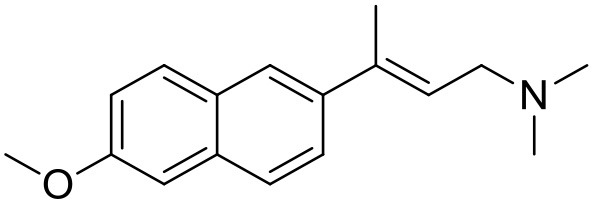	7.67	7.44	Collina et al., 2007
7	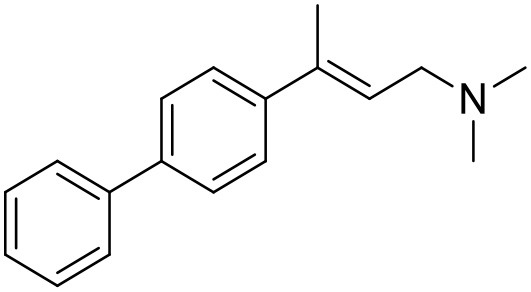	8.85	8.73	Rossi et al., 2011
8^a^	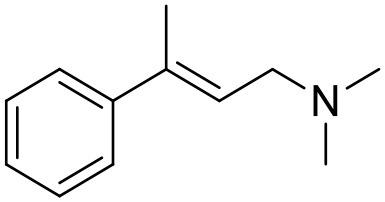	6.00	6.79	Rossi et al., 2011
9	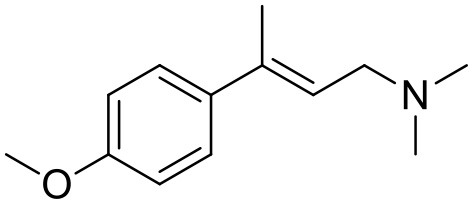	6.09	6.23	Rossi et al., 2011
10	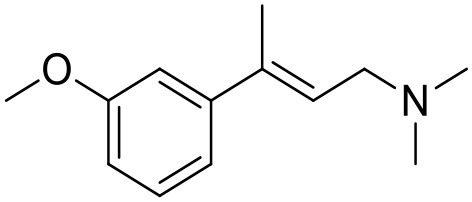	6.00	6.14	Rossi et al., 2011
11	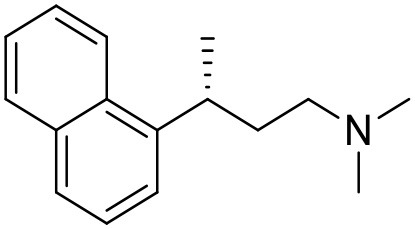	7.33	7.11	Collina et al., 2007
12^a^	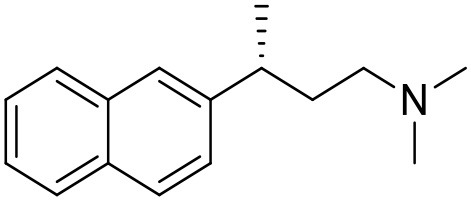	8.71	7.99	Collina et al., 2007
13	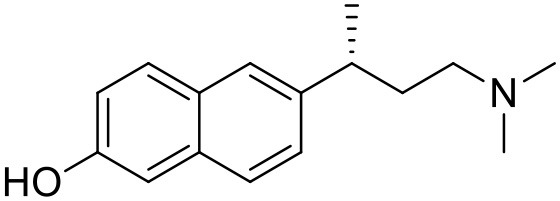	7.72	7.65	Collina et al., 2007
14	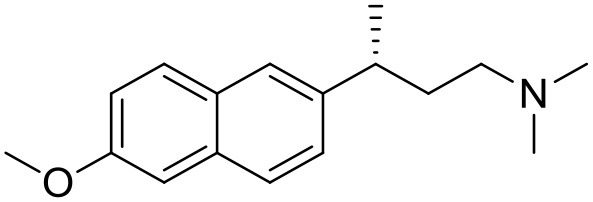	8.64	8.41	Collina et al., 2007
15	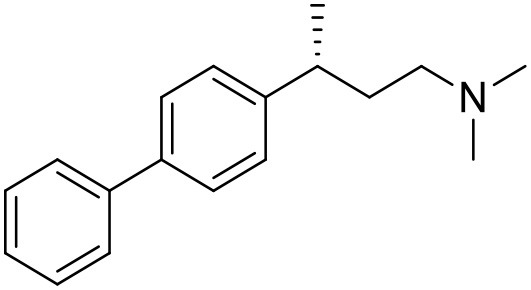	8.99	9.05	Collina et al., 2007
16	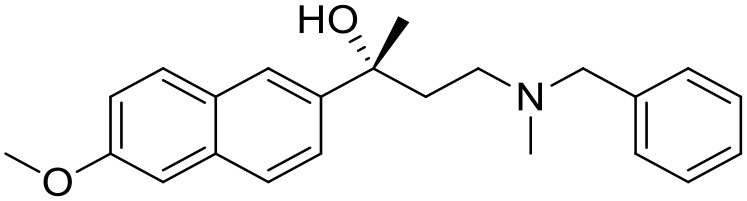	8.22	8.10	Rossi et al., 2010
17	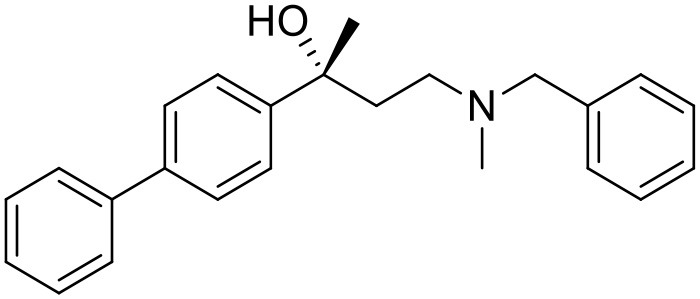	8.62	8.26	Rossi et al., 2010
18	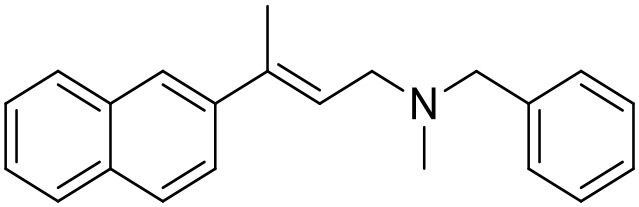	8.10	8.09	Collina et al., 2007
19	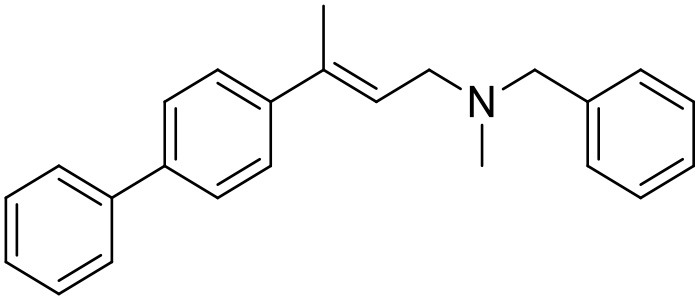	8.20	8.29	Collina et al., 2007
20	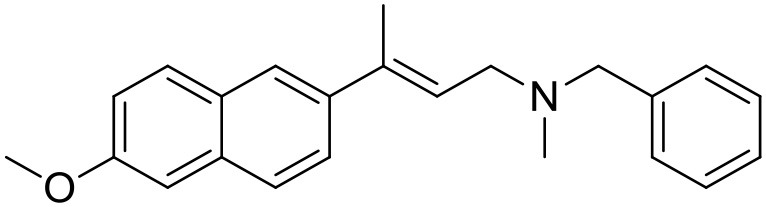	7.86	8.22	Rossi et al., 2010
21	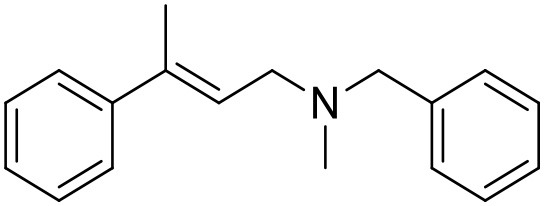	7.04	7.06	Collina et al., 2007
22	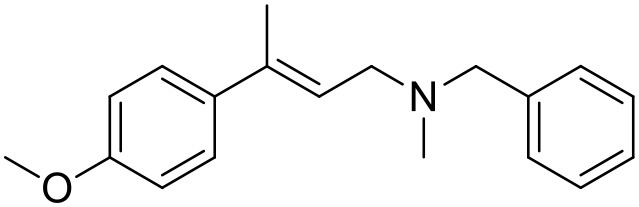	8.28	8.11	Collina et al., 2007
23^a^	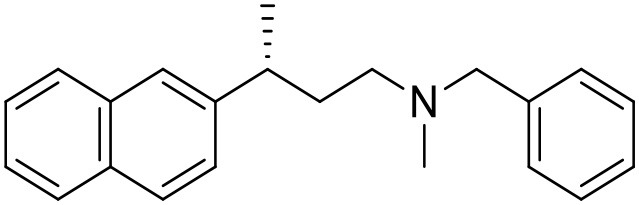	8.27	8.96	Collina et al., 2007
24	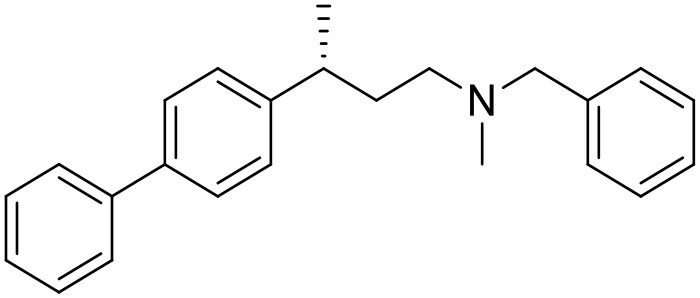	8.24	8.30	Collina et al., 2007
25	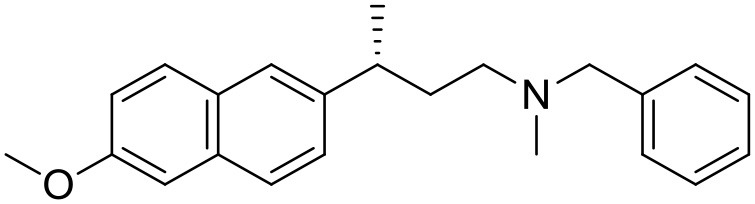	8.64	8.52	Rossi et al., 2010
26	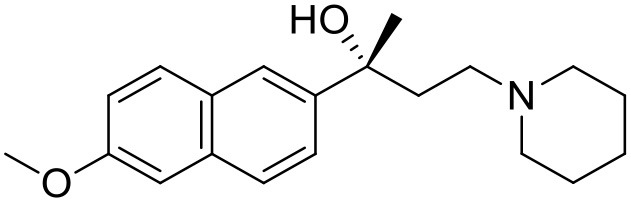	7.98	8.01	Rossi et al., 2010
27	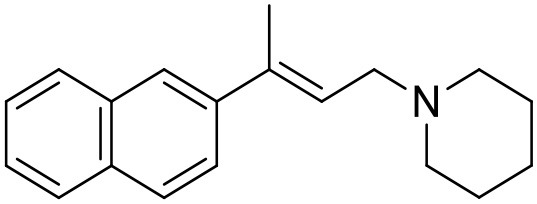	9.01	8.79	Rossi et al., 2011
28	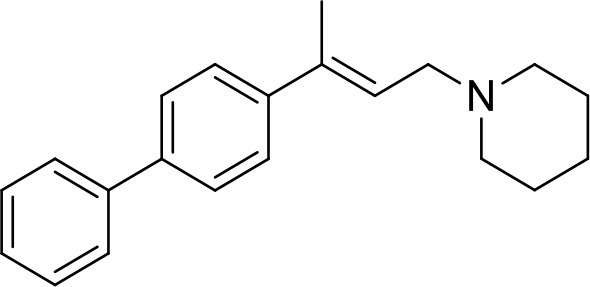	9.07	9.22	Rossi et al., 2011
29^a^	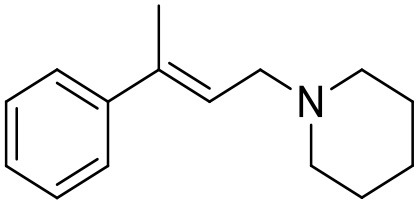	6.34	6.23	Rossi et al., 2011
30	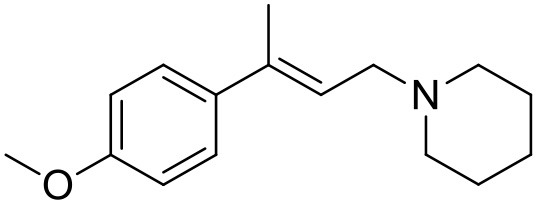	7.85	7.67	Rossi et al., 2011
31	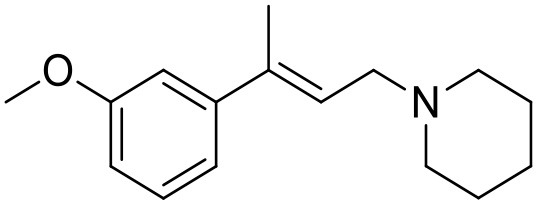	7.04	7.10	Rossi et al., 2011
32	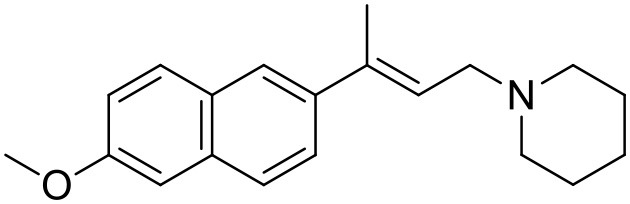	8.36	8.62	Rossi et al., 2010
33	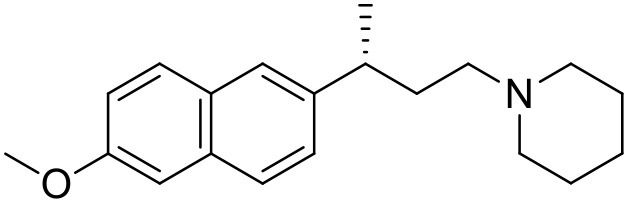	8.89	8.99	Rossi et al., 2010
34^a^	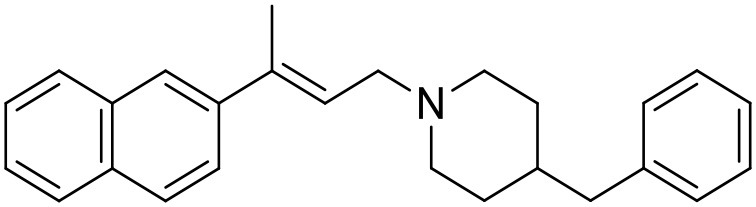	7.64	7.65	Rossi et al., 2011
35	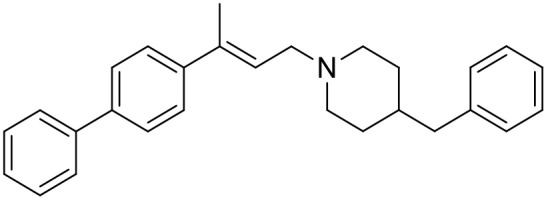	8.15	8.20	Rossi et al., 2011
36^a^	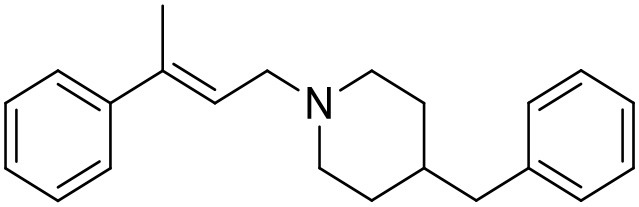	8.97	8.56	Rossi et al., 2011
37^a^	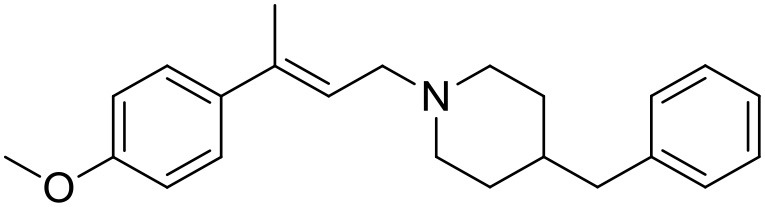	8.38	8.28	Rossi et al., 2011
38^a^	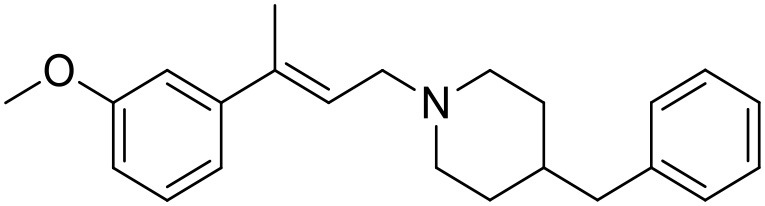	8.11	7.40	Rossi et al., 2011
39	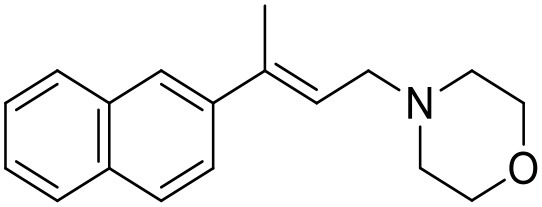	8.02	8.04	Rossi et al., 2011
40	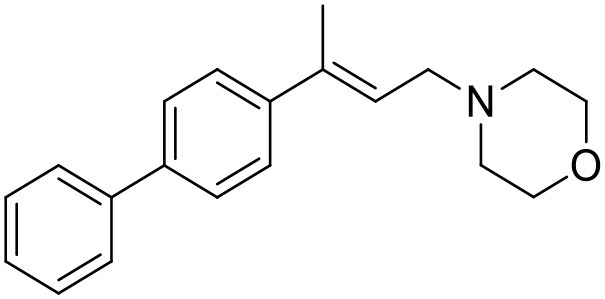	7.94	7.83	Rossi et al., 2011
41	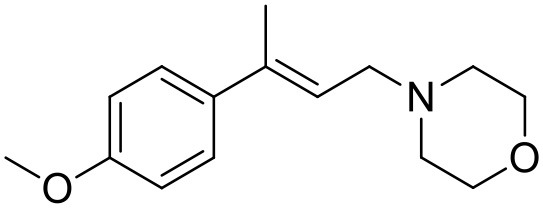	6.00	6.38	Rossi et al., 2011
42	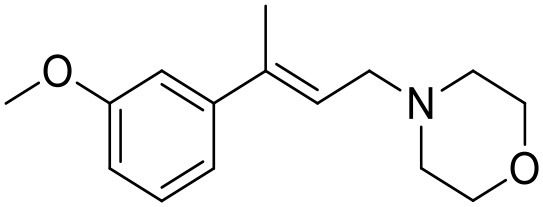	6.00	5.98	Rossi et al., 2011
43^a^	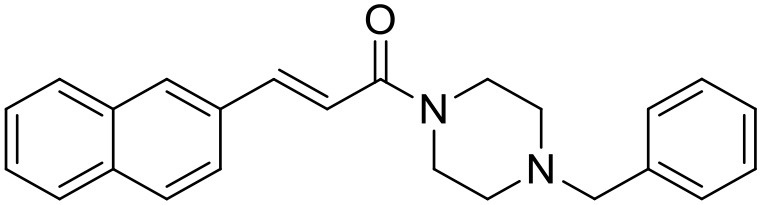	6.00	7.29	Urbano et al., 2007
44	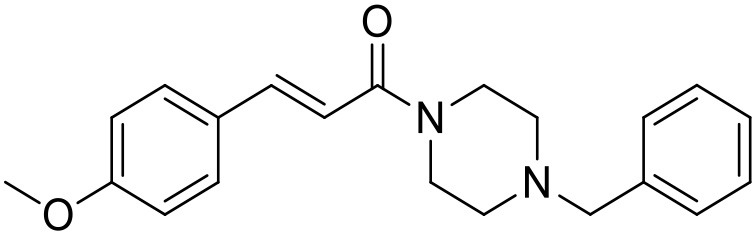	6.00	6.03	Urbano et al., 2007
45	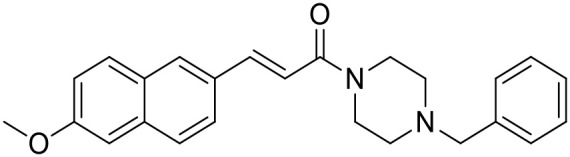	6.00	6.16	Urbano et al., 2007
46	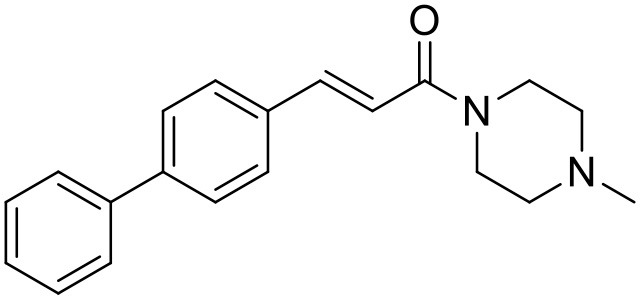	6.00	5.78	Urbano et al., 2007
47^a^	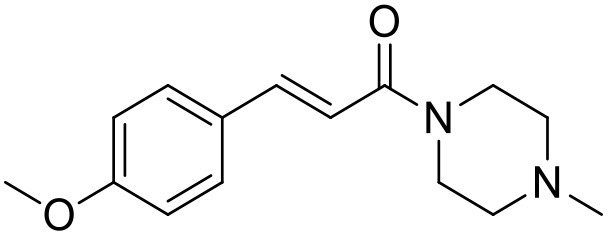	6.00	6.36	Urbano et al., 2007
48	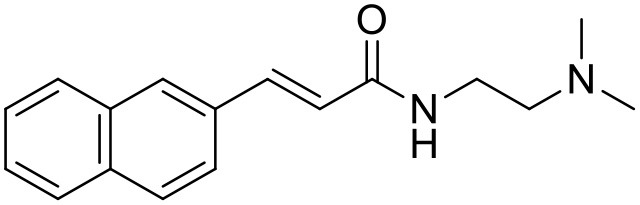	6.00	5.89	Urbano et al., 2007
49	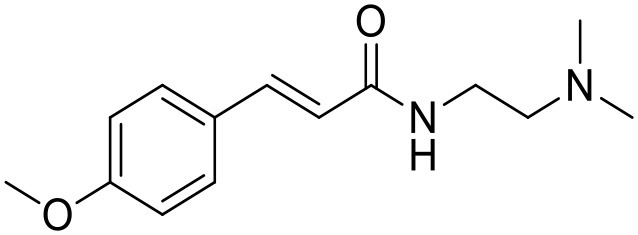	6.00	5.75	Urbano et al., 2007
50	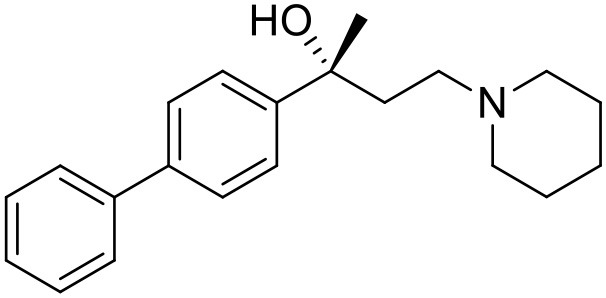	7.41	7.52	Rossi et al., 2015
51	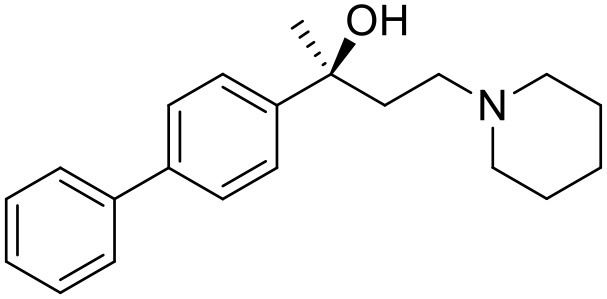	8.33	8.37	Rossi et al., 2015
52	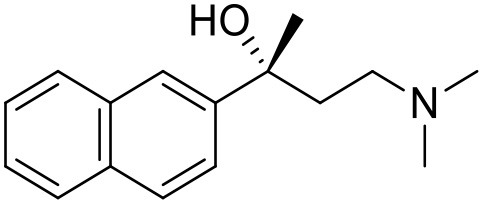	6.69	6.81	Rossi et al., 2015
53	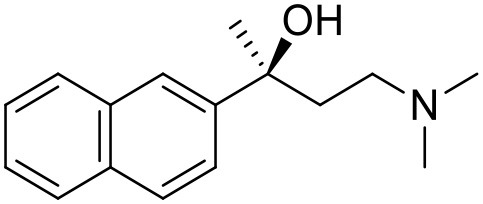	7.20	7.40	Rossi et al., 2015
54^a^	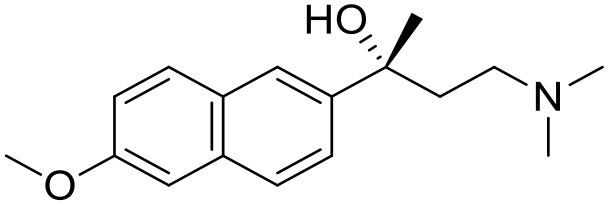	7.29	7.44	Rossi et al., 2015
55	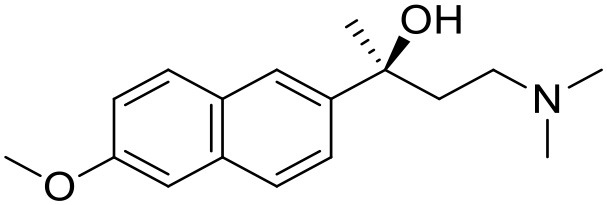	7.60	7.90	Rossi et al., 2015
56	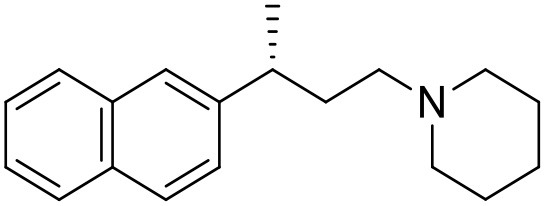	8.82	8.57	Rossi et al., 2017
57	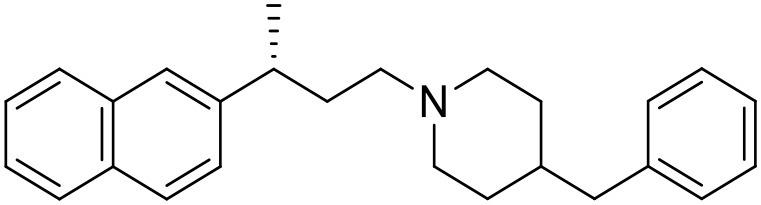	8.22	8.29	Rossi et al., 2017
58	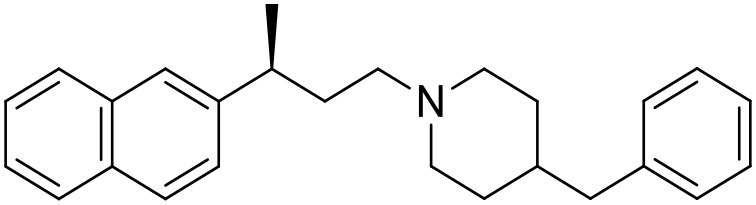	8.16	8.14	Rossi et al., 2017
59	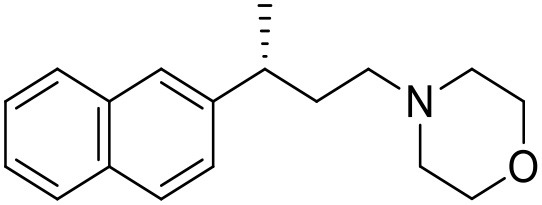	8.27	8.20	Rossi et al., 2017
60	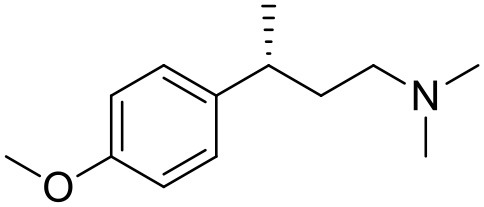	6.94	6.91	Rossi et al., 2017
61	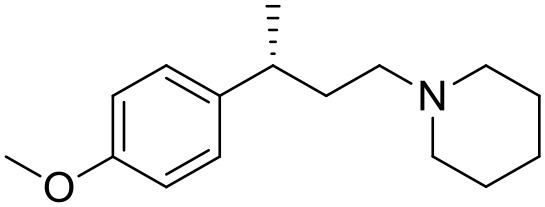	7.70	7.68	Rossi et al., 2017
62	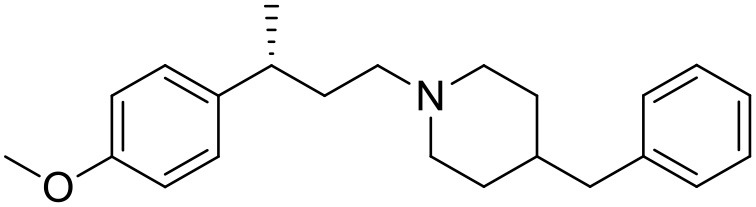	8.46	8.37	Rossi et al., 2017
63	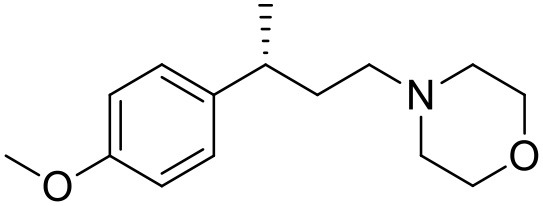	7.12	7.20	Rossi et al., 2017
64	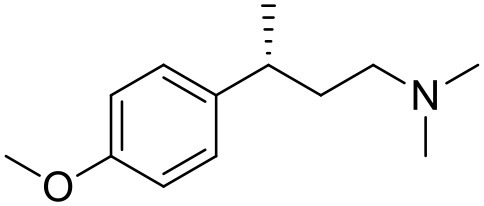	6.62	6.75	Rossi et al., 2017
65^a^	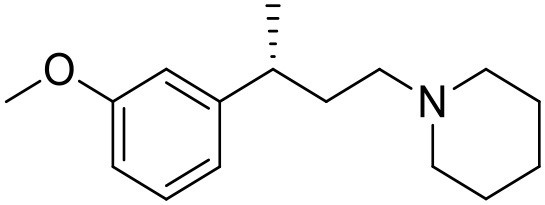	7.44	6.42	Rossi et al., 2017
66	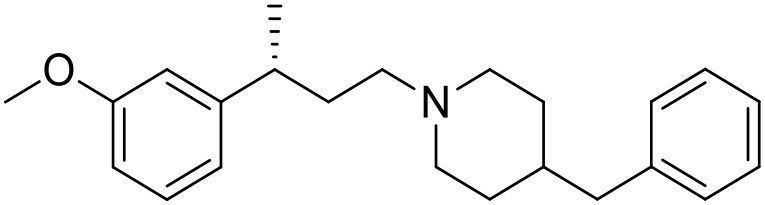	8.54	8.56	Rossi et al., 2017
67^a^	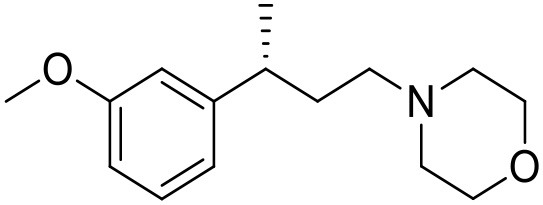	6.86	7.25	Rossi et al., 2017
68	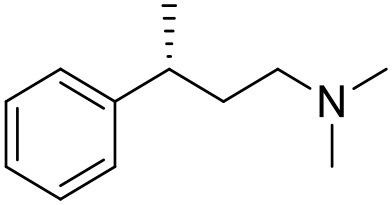	6.37	6.35	Rossi et al., 2017
69	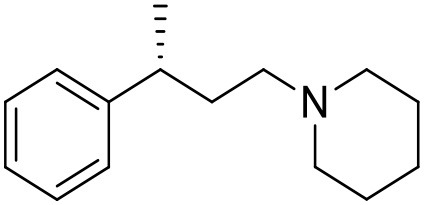	7.34	7.37	Rossi et al., 2017
70^a^	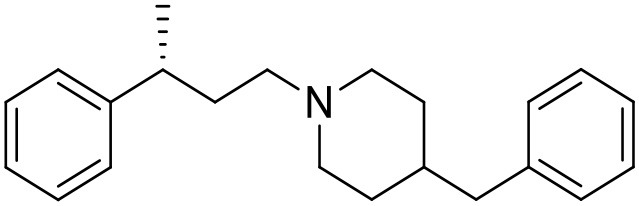	8.54	8.62	Rossi et al., 2017
71	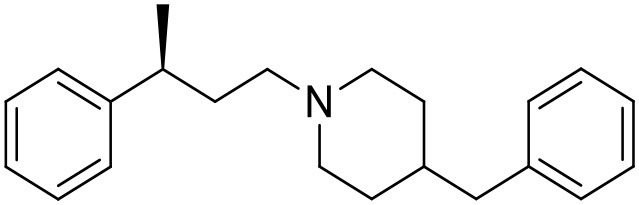	8.52	8.45	Rossi et al., 2017
72^a^	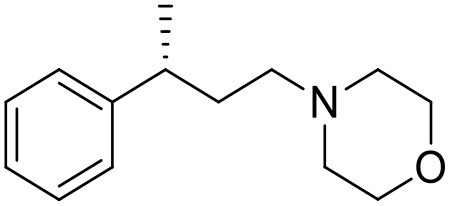	7.07	6.56	Rossi et al., 2017
73	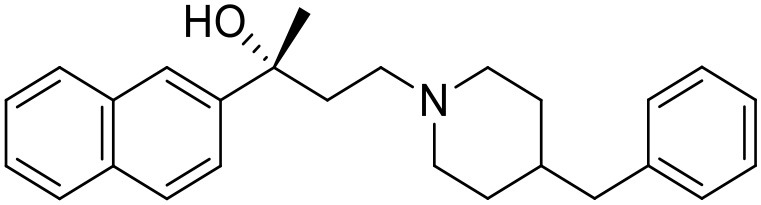	8.00	8.01	Rui et al., 2016
74	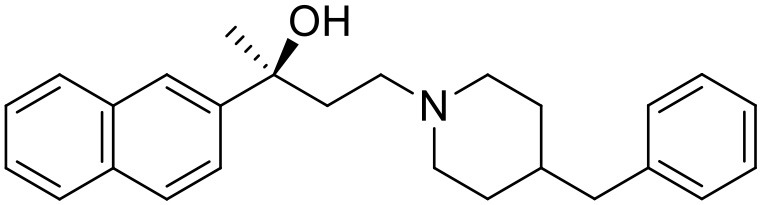	7.96	7.92	Rui et al., 2016
75	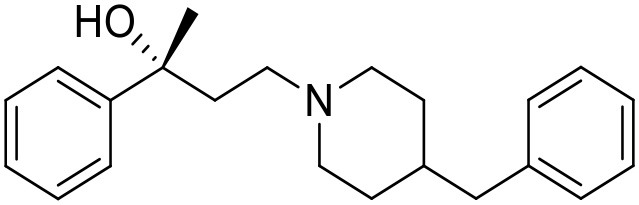	7.57	7.73	Rui et al., 2016
76	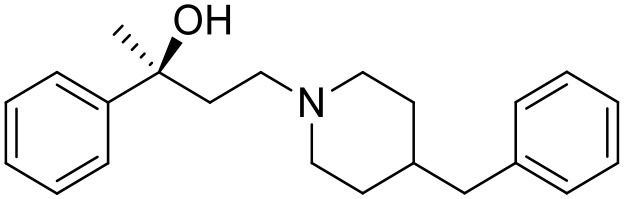	7.40	7.45	Rui et al., 2016
77	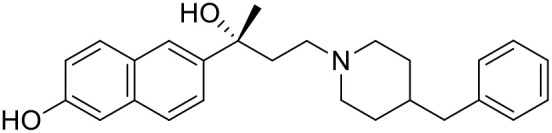	7.15	7.09	Rui et al., 2016
78	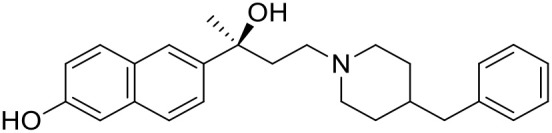	7.21	7.18	Rui et al., 2016
79	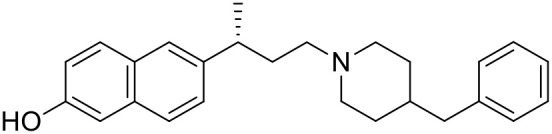	7.46	7.42	Rui et al., 2016
80^a^	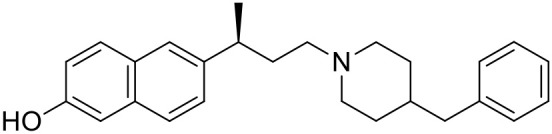	7.89	7.19	Rui et al., 2016

a *Test set compounds*.

b *Experimental and predicted pKi values using Model SE*.

### Molecular Docking

The ligand-receptor molecular docking experiments of RC-33 analogs into the active site of S1R were performed by using the software Glide from the Schrödinger suite (Friesner et al., 2004). Glide is one of the most effective docking programs at this moment with many successful applications relating to rational design of novel drugs and investigation of protein-ligand interactions. Such applications encompass *in silico* search of novel drugs (Osguthorpe et al., 2012; Amaning et al., 2013), analysis of the SAR of congeneric series of compounds (Almerico et al., 2012; Quesada-Romero and Caballero, 2014; Quesada-Romero et al., 2014; Mena-Ulecia et al., 2015), evaluation of enzymatic reaction pathways (Wu et al., 2011; Batra et al., 2013), etc.

Protein coordinates were extracted from the crystal structure of S1R bound to the selective antagonist PD144418 (code 5HK1 in Protein Data Bank) (Schmidt et al., 2016). A grid box of 20 × 20 × 20Å was centered on the center of mass of the ligand in this crystal structure covering the binding site of S1R. Glide standard (SP) and extra-precision (XP) modes were employed with the same protocol and parameters that were used by us in previous works (Quesada-Romero and Caballero, 2014; Quesada-Romero et al., 2014; Mena-Ulecia et al., 2015). Glide SP was used to evaluate the capability of the Glide method to obtain poses that fit the known pharmacophore of S1R ligands, and the more precise Glide XP was used for finding the final docking poses.

After several poses were found for each compound, the ones that showed the best scoring energies were considered. The information of PD144418, 4-IBP, haloperidol, NE-100, and (+)-pentazocine in the crystallographic structures recently reported (Schmidt et al., 2016, 2018) was considered for the selection of the best solutions; these compounds show how the previously reported pharmacophoric pattern (Glennon, 2005) is oriented inside the S1R binding site. The essential chemical interactions described for analog ligands (ECIDALs) (Muñoz-Gutierrez et al., 2016; Ramírez and Caballero, 2018) defined for S1R ligands were identified using this information. The most obvious essential chemical interaction is that charged amino group of the ligands must be close to the side chain carboxylate group of the residue Glu172, forming an electrostatic interaction. Therefore, the best docking solution for each compound was the pose that had the best scoring energy and complies with this essential chemical interaction.

The “Interaction Fingerprints Panel” of Maestro (Maestro 10.2.011, Schrödinger LLC) was used for deriving the Interaction fingerprints (IFPs) as described in Singh et al. reports (Deng et al., 2004; Singh et al., 2006). The method accounts for the presence of different types of chemical interactions between ligands and the binding site residues of the target receptor by using bits. For this purpose, distance cutoffs are defined for the binding site, and the interacting set encompasses the residues that contain atoms within the specified cutoff distance from ligand atoms. An interaction matrix is constructed including the bits with relevant information of the defined chemical interactions.

### QSAR Modeling

After docking experiments, 3D-QSAR models were performed to explain the SAR of the RC-33 analogs. Their bioactive conformations predicted by using docking were used as the alignment rule for deriving the models. The structural features that affect their activities against the S1R were identified by describing steric and electrostatic fields.

The 80 compounds dataset was randomly partitioned into training (64 compounds) and external (16 compounds) sets. A homogenous distribution of the activities was granted in both training and test sets. 3D-QSAR models were generated using Open3DQSAR (Tosco and Balle, 2011), an open access tool with all the capacities to construct 3D-QSAR models. Steric and electrostatic fields were computed according to classical molecular mechanics equations using the Merck Molecular Force Field (Halgren, 1996).

The field variables were calculated by describing the interaction energies between probe atoms (sp^3^ carbon atoms with a charge +1) and structures in a 1.0 Å step size grid box surrounding the whole set. Variables were processed as follows: (i) high energies adopted the top value of 30 kcal/mol, (ii) energy values very close to zero (below 0.05 kcal/mol) were set to zero in order to reduce noise, (iii) variables which only assumed a few different values (*n*-level variables) were removed. Thereafter, variables were scaled using the Block Unscaled Weighting procedure (Kastenholz et al., 2000; Boháč et al., 2002) and the predictive power of the models was improved by using the Smart Region Definition algorithm (Pastor et al., 1997).

Partial Least Square (PLS) regression was used to construct 3D-QSAR models, including from one to five Principal Components (PCs) and different combinations of fields. Models were derived by using one field and by combining them; the best model was selected by considering the higher value of the internal leave-one-out (LOO) cross-validation *Q*^2^.

## Results and Discussion

### Docking Predictions

We have a structural information of the binding poses of S1R ligands such as PD144418, 4-IBP, haloperidol, and NE-100 that similar in shape to RC-33. This information was used for evaluating the quality of the obtained docking results for RC-33 and its analogs. It is known that S1R ligands contain a charged nitrogen central atom flanked by two hydrophobic regions of different size (Glennon, 2005). The above mentioned S1R ligands form electrostatic interactions between the ligand charged nitrogen atoms and the side chain carboxylate of Glu172. In addition, their larger hydrophobic groups locate near the residues Val84, Met93, Leu95, Leu105, Tyr206, Ile178, Leu182, and Tyr103 (primary hydrophobic site), and their smaller hydrophobic groups locate near the residues Phe107, Trp164, His154, and Ile124 (secondary hydrophobic site). It is expected that the studied compounds establish such interactions.

**Figure 1:**
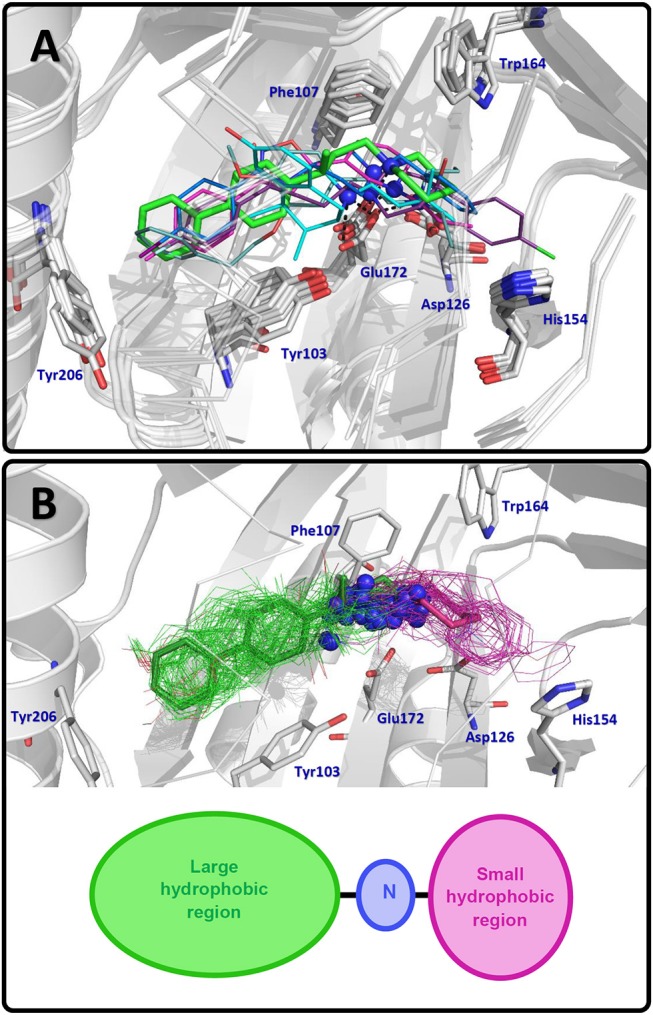
Docking results for RC-33 and its analogs. **(A)** Docking pose obtained for RC-33 (stick representation in green) and comparison with X-ray crystallographic structures of the antagonist PD144418 (thin stick representation in purple, PDB code 5HK1), the ambiguous ligand 4-IBP (thin stick representation in light blue, PDB code 5HK2), the antagonist haloperidol (thin stick representation in lilac, PDB code 6DJZ), the antagonist NE-100 (thin stick representation in teal, PDB code 6DK0), and the agonist (+)-pentazocine (thin stick representation in cyan, PDB code 6DK1). N positively charged atom for each compound is represented by a blue sphere. **(B)** (top) Docking of RC-33 (in sticks representation) and comparison with its analogs (in lines representation); for each compound large hydrophobic group is in green at the left, small hydrophobic group is in purple at the right, and N positively charged atom is a sphere in blue. (bottom) Pharmacophoric model for S1R ligands: N positively charged atom (blue) flanked by large hydrophobic (green) and small hydrophobic (purple) regions.

Docking orientations of RC-33 and its analogs are represented in Figure 1. The best docking pose obtained for RC-33 was compared with the orientations of PD144418, 4-IBP, haloperidol, NE-100, and (+)-pentazocine in the reference crystallographic structures 5HK1, 5HK2, 6DJZ, 6DK0, and 6DK1, respectively. (+)-Pentazocine is an agonist as RC-33, but it is shorter than RC-33 and the other crystallized ligands; therefore, it is the least suitable ligand for the structural comparison between the crystallized ligands and the docked RC-33 analogs. Figure 1A shows that the docked structure of RC-33 was similarly oriented as the other crystallized ligands. On the other hand, Figure 1B shows that suitable binding modes of the ligands were found for all the RC-33 analogs. All of them form the conserved salt bridge between the charged N atom of the ligands and the residue Glu172 of the S1R. They also oriented their large hydrophobic groups to the primary hydrophobic site, and oriented their small hydrophobic groups to the secondary hydrophobic site. Representations in Figure 1 show that our docking poses are similar to the S1R-ligand X-ray structures reported to date.

We calculated RMSD values for the studied compounds with respect to the docking result of RC-33 inside the S1R by using an *in-house* script (Velázquez-Libera et al., 2018). These calculations show the similarity in orientations between RC-33 and its analogs in an easy way. Since the RC-33 derivatives are different from the reference compound, RMSD values were calculated by considering only the common graphs between molecules. %RefMatch and %MolMatch values were defined, where %RefMatch refers to the percent of common graphs between the docked compound and RC-33 regarding the total number of atoms of RC-33; meanwhile, %MolMatch refers to the percent of common graphs between the docked compound, and RC-33 regarding the total number of atoms of the docked compound. These values allow identifying the maximal similitude between the docked compound and RC-33; therefore, an RMSD value with high %RefMatch and %MolMatch values reflects that the compound under analysis bears a strong resemblance with RC-33.

The majority of the compounds under study have the 1-(3-phenylbutyl)piperidine or parts of this group in common with RC-33. Their RMSD values are reported in Table 2. In general, RMSD values reflect that the majority of compounds had the 1-(3-phenylbutyl)piperidine (or part of this group) similarly oriented with respect to RC-33 (RMSD < 2 Å). However, Table 2 reports compounds with RMSD > 2.5 Å (for instance, compounds **11** (R and S), **57**, **60** (S), and **77**). The 1-(3-phenylbutyl)piperidine group of these compounds is displaced toward the helices α4 and α5; however, their amine groups keep the salt bridge interaction with the residue Glu172. In addition, we found in Table 2 compounds with RMSD > 4 Å (for instance, compounds **37**, **62** (S), **66** (S), **75**, and **76**). The 1-(3-phenylbutyl)piperidine group of these compounds is oriented to the reverse with respect to this group in RC-33; their amine groups also keep the salt bridge interaction with the residue Glu172. These compounds have larger hydrophobic substituents at position 4 of the piperidine, increasing the size of this group. The changed groups fit better inside the bigger hydrophobic cavity close to the helices α4 and α5 when their orientations are opposed to the orientation of the 1-(3-phenylbutyl)piperidine group in RC-33. In this way, these compounds are also adapted to the previous described pharmacophore pattern for S1R ligands (Ablordeppey et al., 2000; Glennon, 2005; Caballero et al., 2012) (the N positively charged atom flanked by two hydrophobic groups of different sizes), where the charged atom is salt-bridged to Glu172, the bigger hydrophobic group is placed near the helices α4 and α5 at the membrane proximal, and the smaller hydrophobic group is placed near the narrow end of the cupin barrel that is further from the membrane.

**Table 2:** RMSD values of the obtained docking pose common fragments for the studied compounds with respect to the docking result of RC-33 inside the S1R.

**ID**	**RMSD (Å)^a^**	**%RefMatch^b^**	**%MolMatch^c^**	**RMSD (Å)^a^,^d^**	**%RefMatch^b^,^d^**	**%MolMatch^c^,^d^**
1 (RC-33)				0.86	100	100
2	1.70	50	61	2.30	50	61
3	1.40	50	55	1.82	50	55
4	2.61	36	47			
5	2.20	36	47			
6	1.42	36	42			
7	0.46	64	74			
8	2.94	36	62			
9	1.67	36	53			
10	2.50	36	53			
11	2.80	50	65	2.89	50	65
12	2.05	50	65	2.16	50	65
13	2.21	50	61	1.50	50	61
14	1.43	50	58	1.46	50	58
15	0.43	77	89	0.86	77	89
16	1.45	50	42	1.07	50	42
17	0.97	77	65	0.69	77	65
18	1.58	36	35			
19	0.42	64	56			
20	1.29	36	32			
21	2.85	36	42			
22	0.38	36	38			
23	2.09	50	48	1.94	50	48
24	0.94	77	68	0.83	77	68
25	1.22	50	44	0.82	50	44
26	1.23	73	70	1.98	73	70
27	1.38	36	40			
28	0.59	64	64			
29	2.24	36	50			
30	0.85	36	44			
31	1.15	36	44			
32	1.11	36	36			
33	2.15	73	73	1.78	73	73
34	2.14	36	30			
35	0.45	64	48			
36	3.70	36	35			
37	4.17	36	32			
38	2.41	36	32			
39	1.16	36	40			
40	0.63	64	64			
41	0.77	36	44			
42	0.72	36	44			
43	2.10	32	26			
44	1.10	32	28			
45	1.66	32	24			
46	0.67	59	57			
47	1.05	32	37			
48	2.28	32	35			
49	2.23	32	39			
50	0.96	100	96			
51	0.64	100	96			
52	1.50	50	61			
53	1.87	50	61			
54	1.48	50	55			
55	1.07	50	55			
56	2.19	73	80	1.96	73	80
57	2.63	73	59			
58	2.22	73	59			
59	1.95^e^	91	100	1.98^e^	91	100
60	1.80	50	73	2.54	50	73
61	2.63	73	89	1.52	73	89
62	3.02	73	64	6.97	73	64
63	2.38^e^	82	100	2.51^e^	82	100
64	2.11	50	73	2.58	50	73
65	2.45	73	89	1.01	73	89
66	2.68	73	64	4.26	73	64
67	2.19^e^	82	100	1.98^e^	82	100
68	0.63	50	85	2.96	50	85
69	2.65	73	100	2.73	73	100
70	3.87	73	70			
71	6.66	73	70			
72	2.58^e^	73	100	1.49^e^	73	100
73	2.29	73	57			
74	2.20	73	57			
75	4.65	73	67			
76	4.39	73	67			
77	2.72	73	55			
78	2.34	73	55			
79	1.74	73	57			
80	2.08	73	57			

a *RMSD values considering only the common chemical fragments between the docked compound and the reference compound RC-33*.

b *%RefMatch refers to the percent of common graphs between the docked and reference compound RC-33 concerning the total number of atoms of the reference compound RC-33*.

c *%MolMatch refers to the percent of common graphs between the docked and reference compound RC-33 regarding the total number of atoms of the docked compound*.

d *RMSD, %RefMatch, and %MolMatch values for the S enantiomer of the compounds reported as racemic pairs*.

e *In this case, difference in ring heavy atoms were not considered between the docked compound and the reference compound RC-33*.

The chemical interactions between the RC-33 analogs and the residues at the S1R binding site can be described in detail by using IFPs. This method has been commonly used for identifying the relevant residues involved in protein-ligand affinities (Caballero et al., 2018; Navarro-Retamal and Caballero, 2018; Velázquez-Libera et al., 2018). IFPs capture and label the chemical contacts between a target protein and a set of its ligands as a whole. The chemotypes are identified with the following labels: P (polar groups), H (hydrophobic groups), A (hydrogen bonds where the residue is the acceptor), D (hydrogen bonds where the residue is the donor), Ar (aromatic groups), and Ch (electrostatic interactions with charged groups). IFPs also differentiate between contacts with backbone and contacts with side-chain functional groups. We calculated IFPs by considering the S1R-ligand complexes formed by our docked structures.

**Figure 2:**
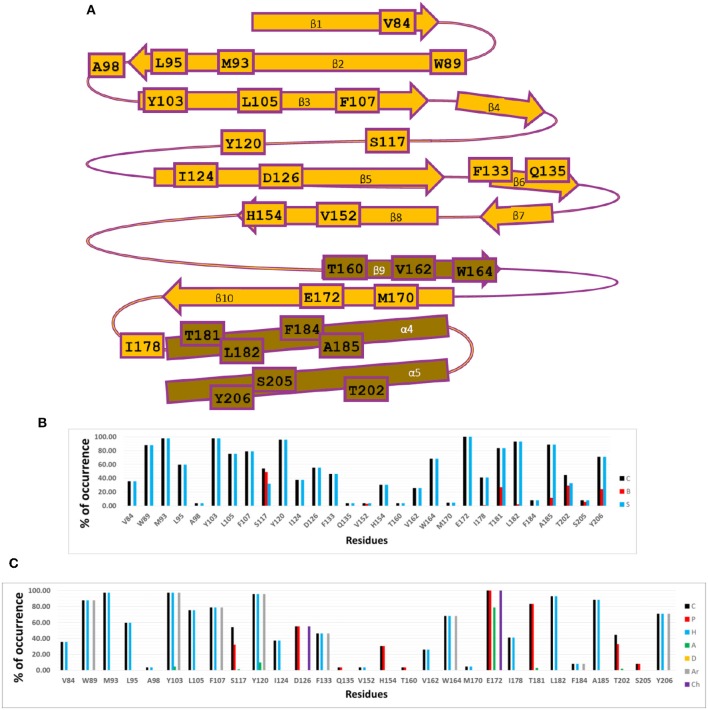
Occurrence of interaction types at the S1R–ligand binding interface. **(A)** Residues with observed interactions, their position in the S1R sequence. **(B)** Percentages of occurrence of contacts C, interactions with the backbone of the residue B, and interactions with the side chain of the residue S. **(C)** Percentages of occurrence of chemical interactions: contacts C, polar P, hydrophobic H, HBs where the residue is acceptor A, HBs where the residue is donor D, aromatic Ar, and electrostatic with charged groups Ch. The S1R–ligand structures obtained by docking were used for calculations of the percentages of occurrence represented here.

The calculated IFPs are reported in Figure 2. The IFP analysis applied to the complexes between S1R and the RC-33 analogs obtained by docking revealed that 29 S1R residues had contacts with ligands. These residues and their positions in the S1R secondary structure are depicted in Figure 2A. The S1R binding site is mainly hydrophobic; in fact, the vast majority of the observed interactions are hydrophobic or aromatic when analyzing the occurrence of chemical contacts in the studied structures (Figure 2C).

The residues with polar interactions were identified in the plots of percent of occurrence obtained from IFP calculations (Figure 2). The residue E172 at the sheet β10 has polar contributions in 100% of the total structures, forming a salt-bridge and it also acts as HB acceptor in 80% of the studied structures. The residue D126 at the sheet β5 was identified with polar contributions in more than 50% of the studied structures. The residue T181 at the helix α4 has polar contributions in more than 80% of the studied structures. Finally, the residues S117 (backbone and side chain), H154 (side chain), and T202 (backbone and side chain) have polar contributions in around 30% of the studied structures.

Several residues with aromatic interactions were also identified in the plots of percent of occurrence obtained from IFP calculations (Figure 2). The residues with aromatic interactions were important for the shape of the S1R binding site because they restrict the space of the pockets. Four aromatic residues located at the center of the binding site (W89, Y103, F107, and Y120) were identified by the IFP calculations with percent of occurrence values above 80%. These residues cause a bottleneck just in front of the residue E172; therefore they could help to orient the positively charged N of the ligands to form the salt bridge. At the same time, they could stabilize the presence of the positive charge by means of π-cation interactions. The aromatic residues F133 at the sheet β6 and W164 the sheet β9, located close to the narrower end of the cupin β-barrel, have percent of occurrence values of 50 and 70%, respectively. On the other hand, the residue Y206, located at the helix α5, has a percent of occurrence value of 70%.

The remaining residues with hydrophobic interactions were also identified in the plots of percent of occurrence obtained from IFP calculations (Figure 2). The residues identified with percent of occurrence above 75% M93 (at β2), L105 (at β3), and L182/A185 (at α4) are located at the bigger hydrophobic pocket. The residues V84 (at β1), L95 (at β2), and I178 (at the loop between β10 and α4) are also located at the bigger hydrophobic pocket and were identified by IFP calculations with lower percent of occurrences, and the residue I124 at β5, located at the smaller hydrophobic pocket, had a percent of occurrence below 40%.

In general, the reported IFPs identify the most important S1R residues which establish chemical interactions with RC-33 analogs. Furthermore, it could be useful for the understanding of the interactions between S1R and its ligands.

### 3D-QSAR Results

We constructed the 3D-QSAR models based on docking alignment; therefore, the docked structures were included in a box for creating the relevant fields, since they are models of the ligand conformations inside the S1R binding site. The docking-based or receptor-guided alignment 3D-QSAR is a well-documented method in literature (Guasch et al., 2012; Navarro-Retamal and Caballero, 2016; Muñoz-Gutiérrez et al., 2017). Three 3D-QSAR models were trained using the steric field (Model S), the electrostatic field (Model E), and the combination of both fields (Model SE). The most reliable models were selected by measuring the LOO cross-validation performance (*Q*^2^ > 0.5) and the test set predictions ($R_{\text{test}}^{2}$ > 0.5).

Table 3 lists the description and statistical information of the best 3D-QSAR models. This report proved that model S has better (LOO) cross-validation *Q*^2^ than model E. However, when both steric and electrostatic fields are tied together in the more complex model SE, the Q^2^ value increases; therefore, this model, which had a Q^2^ = 0.70 including seven components, containing a major contribution of the steric field (88%), was identified as the model best describing the structure-activity relationship of the studied RC-33 analogs. These results reflect that the steric features are mandatory for modulating the agonistic activities of the studied compounds. This is reasonable considering that the S1R binding site is mostly hydrophobic.

**Table 3:** Statistical information of the 3D-QSAR models.

**Fields**	**NC**	***R*^**2**^**	***S***	***Q*^**2**^**	***S*_**LOO**_**	**$R_{\text{test}}^{2}$**	***S*_**test**_**	**%S**	**%E**
S	8	0.98	0.13	0.64	0.54	0.34	0.81	1	
E	4	0.81	0.39	0.54	0.62	0.59	0.63	–	1
SE	7	0.97	0.15	0.70	0.50	0.61	0.62	0.88	0.12

*NC is the number of components; S is the standard deviation of the fitted activity of the training set; R^2^, Q^2^, and $R_{test}^{\text{2}}$ are the coefficients of correlation of the training set, LOO cross validation, and test set, respectively; S_LOO_ is the standard deviation of the LOO cross validation, and S_test_ is the standard deviation of the test predictions. %S and %E are the relative contributions of the steric (S) and the electrostatic (E) fields, respectively*.

**Figure 3:**
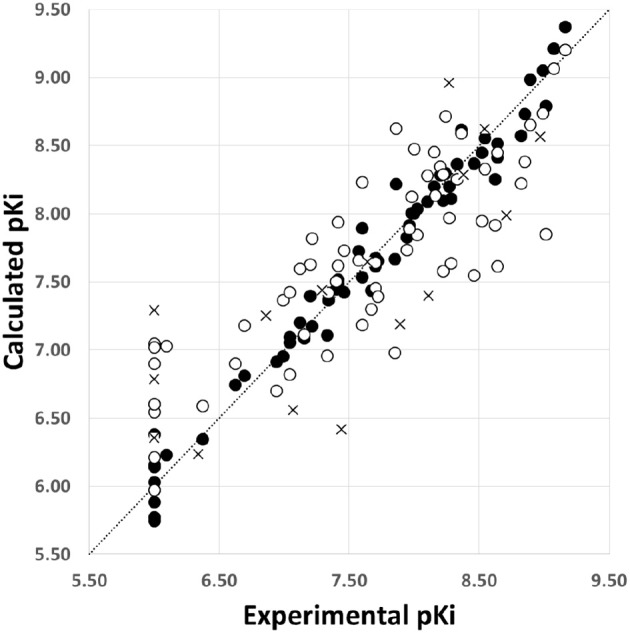
Scatter plot of the experimental activities vs. predicted activities for the model SE: (•) training set predictions, (°) LOO cross-validation predictions, and (×) test set predictions.

The model SE explains 97% of the variance and has a low standard deviation (*S* = 0.15). The predictions of pKi values for the 64 RC-33 analogs in the training set using the model SE are reported in Table 1 and the correlations between the predicted and experimental pKi values (from training and LOO cross validation) are shown in Figure 3. It is possible to observe that the selected model fitted adequately the whole dataset; it is noteworthy that the more potent compounds had an outstanding performance. When the model SE was used to predict the pKi values of the test set compounds, well results were also found, reflected by the value of ${\text{R}}_{test}^{2}$ = 0.61. The predicted pKi values for the test set are listed in Table 1, and the correlation between the calculated and experimental pKi values are plotted in Figure 3.

**Figure 4:**
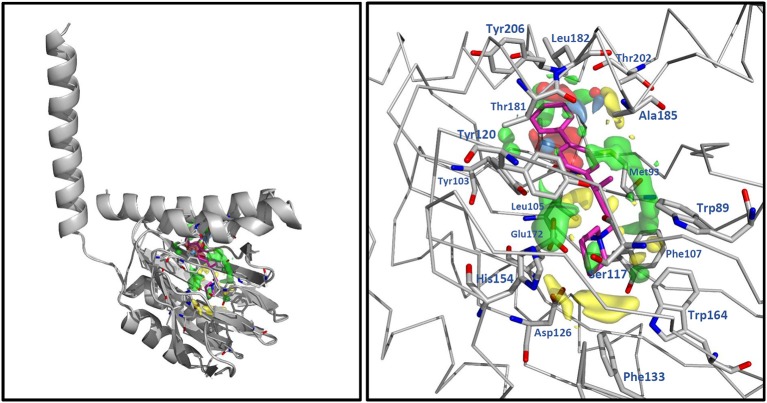
3D-QSAR contour maps for the RC-33 analogs (model SE). The steric field is represented by green and yellow isopleths: the green ones indicate regions where bulky groups enhance the activity, and the yellow ones indicate regions where bulky groups disfavor the activity. The electrostatic field is represented by blue and red isopleths: the blue ones indicate regions where an increase of positive charge enhances the activity and the red ones indicate regions where an increase of negative charge enhances the activity. RC-33 is shown inside the fields.

Figure 4 shows contour plots of the steric and electrostatic fields projected onto the docked structure of RC-33 for association between the fields, the compounds of the whole set, and the residues at the S1R binding site. In this figure, green and yellow contours represent regions with positive and negative steric components, respectively. It is noted that positive steric components have a major role. A great green contour in front of the 3-phenylbutylamine, and near the residues V84, W89, F107, and A185, indicates that bulk groups are desired in this region. It is noteworthy that the most active compounds such as RC-33, **7**, **15**, **27**, **28**, and **33** has the methyl group of the 3-phenylbutylamine in this region, but the majority of the less active compounds such as **8**, **29**, **41**, **64**, and **67** have this group deeper into the bigger hydrophobic pocket. Another three green contours are located near the piperidine of RC-33 and the residues Y120, S117, and W164 indicating that this group or another bulky group in this region is needed. In general, compounds with a dimethylamine in this region (compounds **2**–**15**) are less active than similar compounds that contain piperidine. Another green contour near the residues Y103 and E172 reflects that several active compounds contain the methyl group of the 3-phenylbutylamine in this region. Another green contours are located at the bigger hydrophobic pocket near the residues Y103, Y206, and T202, indicating the preference of a bulky group in this region. For instance, the biphenyl group in compound **7** is preferred instead the phenyl group in compound **8** because the former group fills the entire space of the bigger hydrophobic pocket. Several yellow contours were identified near the residues W164, L105, F107, and T202. All of them are close to the green contours both in the bigger and smaller pockets, and reflect the complexity of the steric field inside the S1R binding site.

In Figure 4, blue and red contours represent regions with positive and negative electrostatic components, respectively; all of them are small and are located inside the bigger hydrophobic pocket. The blue contours are near the residues T181, A185, L182, and the backbone of Y206, and the red contours are near the residues A92, L95, L105, L182, and T202. The blue contours are located in regions where ligands placed hydroxyl groups and their pKi values are between 7 and 7.8 (moderate activities). For instance, compounds **13** and **77** have hydroxyl close to the backbone of Y206, **75**, and **76** have hydroxyl close to A185, and **53**, 55, and **78** have hydroxyl close to T181. The red contours are located in regions where ligands placed OMe groups and the activity is increased. For instance, compounds **22** and **30** that contain OMe have better activities than compounds **21** and **29** without this group.

The docking-based 3D-QSAR methodology allows establishing a comparison between the chemical features that describe the structure-activity relationship of bioactive ligands and the protein binding site (Alzate-Morales and Caballero, 2010; Caballero et al., 2011; Quesada-Romero et al., 2014; Mena-Ulecia et al., 2015; Muñoz-Gutiérrez et al., 2017). The contour plots in receptor-based 3D-QSAR are not receptor maps, but they solve another key point of the description of the differential activities: different potency in activities is connected with different chemical environments and interactions. The docking and 3D-QSAR methods applied to the study of RC-33 analogs give more information about the structure of S1R-ligand complexes, and identify important chemical features to take into account in the future design of potent S1R ligands. We feel that another similar studies on other series of compounds will be reported during next years.

## Conclusion

This is the first structure-based molecular modeling investigation a few years after the elucidation of the S1R crystallographic structure; therefore, details of the binding poses and the chemical interactions in the binding site are described. Binding orientations and structure-activity relationship of RC-33 analogs as S1R agonists were studied by using molecular docking and 3D-QSAR methods.

Docking poses obtained for the studied compounds inside the S1R binding site explain the interactions between the well-known theoretical pharmacophore model reported for these compounds (elucidated before the knowledge of the S1R 3D structure) and the residues located at the binding site. They also reproduced structural features reported for complexes between S1R and PD144418, 4-IBP, and other active ligands. The docking analysis, including the IFP calculations, confirmed the preponderant role of E172 forming a salt bridge with the positively charged N of the ligands. Furthermore, docking experiments also identified the importance role of the aromatic residues delimiting the shape of the S1R binding site: specifically, W89, Y103, F107, and Y120 which are at the center of the binding site, F133 and W164 which are close to the narrower end of the cupin β-barrel, and Y206 which is close to the helix α5.

A receptor-guided alignment 3D-QSAR model with adequate statistical significance and acceptable prediction power was obtained. Steric and electrostatic features had contributions to the differential potency of the agonists, with a major role of the steric ones. The 3D-QSAR model demonstrated that an implicit correlation is found in the data under analysis between the chemical features of the compounds in their active conformations and their interactions in the pockets of the S1R binding site.

Overall, the information reported here, derived from the recently reported S1R structure, will be useful for the future research in the design of novel S1R ligands.

## Data Availability

The raw data supporting the conclusions of this manuscript will be made available by the authors, without undue reservation, to any qualified researcher.

## Author Contributions

The work was completed by cooperation of all authors. JC was responsible for the study of concept and design of the project. JV-L performed the docking RMSD analysis, IFPs, and 3D-QSAR calculations. GR and CN-R performed the docking calculations. SC and JC drafted and revised the manuscript.

### Conflict of Interest Statement

The authors declare that the research was conducted in the absence of any commercial or financial relationships that could be construed as a potential conflict of interest.
